# Organisational change in hospitals: a qualitative case-study of staff perspectives

**DOI:** 10.1186/s12913-019-4704-y

**Published:** 2019-11-14

**Authors:** Chiara Pomare, Kate Churruca, Janet C. Long, Louise A. Ellis, Jeffrey Braithwaite

**Affiliations:** Centre for Healthcare Resilience and Implementation Science, Australian Institute of Health Innovation, Macquarie University, 75 Talavera Rd, Macquarie Park, Australia

**Keywords:** Organisational change, Health systems change, Hospital redevelopment, Hospital expansion, Staff expectations

## Abstract

**Background:**

Organisational change in health systems is common. Success is often tied to the actors involved, including their awareness of the change, personal engagement and ownership of it. In many health systems, one of the most common changes we are witnessing is the redevelopment of long-standing hospitals. However, we know little about how hospital staff understand and experience such potentially far-reaching organisational change. The purpose of this study is to explore the understanding and experiences of hospital staff in the early stages of organisational change, using a hospital redevelopment in Sydney, Australia as a case study.

**Methods:**

Semi-structured interviews were conducted with 46 clinical and non-clinical staff working at a large metropolitan hospital. Hospital staff were moving into a new building, not moving, or had moved into a different building two years prior. Questions asked staff about their level of awareness of the upcoming redevelopment and their experiences in the early stage of this change. Qualitative data were analysed using thematic analysis.

**Results:**

Some staff expressed apprehension and held negative expectations regarding the organisational change. Concerns included inadequate staffing and potential for collaboration breakdown due to new layout of workspaces. These fears were compounded by current experiences of feeling uninformed about the change, as well as feelings of being fatigued and under-staffed in the constantly changing hospital environment. Nevertheless, balancing this, many staff reported positive expectations regarding the benefits to patients of the change and the potential for staff to adapt in the face of this change.

**Conclusions:**

The results of this study suggest that it is important to understand prospectively how actors involved make sense of organisational change, in order to potentially assuage concerns and alleviate negative expectations. Throughout the processes of organisational change, such as a hospital redevelopment, staff need to be engaged, adequately informed, trained, and to feel supported by management. The use of champions of varying professions and lead departments, may be useful to address concerns, adequately inform, and promote a sense of engagement among staff.

## Background

Change is a common experience in complex health care systems. Staff, patients and visitors come and go [1]; leadership, models of care, workforce and governing structures are reshaped in response to policy and legislative change [2], and new technologies and equipment are introduced or retired [3]. In addition to these common changes experienced throughout health care, the acute sector in many countries is constantly undergoing major changes to the physical hospital infrastructure [4, 5]. In New South Wales, Australia, several reports have described the increase in hospital redevelopment projects as a ‘hospital building boom’ [4, 6], with approximately 100 major health capital projects (i.e., projects over AUD$10 million) currently in train [7]. In addition to meeting the needs of a growing and ageing population [8], the re-design and refurbishment of older hospital infrastructure is supported by a range of arguments and anecdotal evidence highlighting the positive relationship between the hospital physical environment and patient [9] and staff outcomes [10]. While there are many reasons why hospital redevelopments are taking place, we know little about how hospital staff prospectively perceive change, and their experiences, expectations, and concerns. Hospital staff encapsulates any employee working in the hospital context. This includes clinical and non-clinical staff who provide care, support, cleaning, catering, managerial and administrative duties to patients and the broader community.

One reason as to why little research has explored the perspectives of hospital staff during a redevelopment may be because hospital redevelopment is often considered a physical, rather than organisational change. Organisational change means that not only the physical environment is altered, but also the behavioural operations, structural relationships and roles, and the hospital organisational culture may transform. For example, changing the physical health care environment can affect job satisfaction, stress, intention to leave [11], and the way staff work together [12].

Redeveloping a hospital can be both an exciting and challenging time for staff. In a recent notable example of opening a new hospital building in Australia, staff attitudes shifted from appreciation and excitement in the early stages of change to frustration and angst as the development progressed [13]. Similar experiences have been reported elsewhere, such as in a study describing the consequences for staff of hospital change in South Africa [14]. However, these examples explored staff attitudes towards change retrospectively and considered the change as a physical redevelopment, rather than organisational change. Such retrospective reports may be limited in validity [15] as prospective experiences and understanding of change reported by staff may be conflated with the final outcome of the change. The hospital redevelopment literature has also prospectively assessed health impacts of proposed redevelopment plans as a means to predetermine the impact of a large change on the population [16]; while prospective, this research again considers redevelopment as a physical modification, rather than an organisational change. Thus, while the literature has reported retrospective accounts of staff experiences in large hospital change and prospective assessment of the impact of the change, there is little research examining the understanding and experiences of staff in the early stages of redevelopmental change in hospitals through a lens of organisational change.

Seminal research in the organisational change literature highlights that the role of frontline workers (in this case hospital staff) is crucial to implementation of any process or change [17, 18]. Specifically, that the support of actors (understanding, owning, and engaging) can determine the success of a change [19]. This is consistent with complexity science accounts which suggest that any improvement and transformation of health systems is dependent upon the actors involved, and the extent and quality of their interactions, their emergent behaviours, and localised responses [1, 20]. In health care, change can be resisted when it is imposed on actors (in this case, hospital staff), but may be better accepted when people are involved and adopt a sense of ownership of the changes that will affect them [21]. This may include being involved in the design process. For this reason, it is important to examine the understanding and experiences of actors involved in a change (i.e., hospital staff in a redevelopment), in order to understand and potentially address their concerns, alleviating negative expectations prior to the change.

### Aims

This study is part of a larger project exploring how hospital redevelopment influences the organisation, staff and patients involved [22]. The present study aimed to explore the understanding and experiences of staff prior to moving into a new building as one stage in a multidimensional organisational change project. The research questions were: How do staff make sense of this organisational change? How well informed do they feel? What are their expectations and concerns? What are the implications for hospitals undergoing organisational change, particularly redevelopment?

## Methods

The study protocol has been published elsewhere [22]. The *Consolidated criteria for reporting qualitative research* (COREQ) guidelines were used to ensure comprehensive reporting of the qualitative study results (Additional file 1) [23]*.*

### Study setting and participants

This study was conducted at a large metropolitan, publicly-funded hospital in Sydney, Australia. The facility is undergoing a multimillion-dollar development project to meet the growing needs of the community. This hospital has undergone a number of other changes over the last two decades, including incremental increases in size. Since its opening in the mid 1990s (with approximately 150 beds), several buildings have been added over the years. The hospital now has multiple buildings and over 500 beds.

During the time of this study, the hospital was in the second stage of the multi-stage redevelopment. This stage included: the opening of a new acute services building, the relocation of several wards to this new building (e.g., Intensive care unit (ICU) and Maternity), increases in resources (e.g., equipment, staffing), and the adoption of new ways of working (e.g., activity-based workspaces for support staff). Essentially, the redevelopment involves the opening of a new state-of-the-art building which will include moving services (and staff) from the old to the new building, with some wards staying in the old building. For the wards moving into the new building, this change does not initially involve more patients in existing services, but is intended to increase the number of staff because there will be more physical space to cover and new models of care introduced (e.g., ICU changing to single-bed rooms, more staff needed to individually attend to patients). The current redevelopment includes space for future expansion to account for the growing population. In addition to the redevelopment of the physical infrastructure, the way staff work together is also planned to change. Hospital leadership is aiming to foster a cultural shift towards greater cohesion and unity; highlighting that the hospital redevelopment can be conceptualised as an organisational change of considerable importance and magnitude.

Participants were hospital staff (clinical and non-clinical) working at the hospital under investigation. Staff working on four wards were targeted for interviews, with the intention to capture diverse experiences of the redevelopment and the broader organisational change; two of these wards would be moving into the new building (ICU and Maternity), one ward was not moving (Surgical), and one ward had moved into a new building two years prior (Respiratory). Interviews were also conducted with staff who held responsibilities across wards (e.g., General Services Department: cleaners, porters). The hospital staff were purposively recruited by department heads and snowballed from participants. Fifty staff members were approached (until data saturation was met) with four refusing to participate because they did not have the time.

### Semi-structured interviews

Semi-structured interviews were conducted in private settings at the participants’ place of work (e.g., ward interview rooms, private offices). In the event a participant was unable to meet the researcher in person, interviews were conducted over the phone. A semi-structured interview guide was created in collaboration with key stakeholders from the hospital under investigation and following a literature review. The guide (Additional file 2) included questions aimed at exploring participants’: (1) understanding of the hospital’s culture and current ways of working; (2) understanding of the redevelopment and other hospital changes; and (3) concerns or expectations about the organisational change. The interviews were audio-taped and transcribed verbatim by the first author who is trained and experienced in conducting semi-structured interviews. No field notes were made during the interview nor were transcripts returned to participants for comment or correction due to the time poor characteristics of the study participants (hospital staff). Participants were informed that the research was part of the first author’s doctoral studies.

### Analysis

Interview data were analysed via thematic analysis [24] using NVivo [25]. This approach followed Braun and Clarke’s (2006) six phases of thematic analysis: familiarise, generate initial codes, develop themes, review potential themes, define and name themes, produce the report. Data were initially read multiple times by the first author, then descriptively and iteratively coded according to semantic features. The analysis included the use of inductive coding to identify patterns driven by the data, together with deductive coding, keeping the research questions in mind. Through examination of codes and coded data, themes were developed. The broader research team (KC, LAE, JCL) were included throughout each stage of the analysis process, with frequent discussions concerning the categorization of codes and themes. This process of having one researcher responsible for the analysis while other researchers then checked and clarified emerging themes throughout contributes to the trustworthiness of the findings [26].

In presenting the results, extracts have been edited minimally to enhance readability, without altering meaning or inference. Where extracts are presented, staff are coded according to their department (G: General – works across several wards; ICU: Intensive care unit; MAT: Maternity ward; RES: Respiratory ward; SUR: Surgical ward) and profession (AD: Administrative staff; CHGTEAM: Change management team staff; DR: Medical staff; GS: General services staff; MW: Midwifery staff; N: Nursing staff; OTH: Other profession).

## Results

Forty-six staff members participated in the semi-structured interviews. Interviews were typically conducted face-to-face (*n* = 41; 89.1%), with five interviews conducted over the phone. No differences were discerned in content between these different mediums. Hospital staff taking part in interviews included those from: nursing and midwifery, medical, general services, administrative, and change management (Table 1). Change management staff are external to the hospital staff, and do not report to hospital executives. Interviews ranged from seven to 33 min in length (*M* = 17 min). Participating staff had worked at the hospital for on average 10.5 years (range 5 months and 30 years).

**Table 1:** Profession of interview participants (*N* = 46)

	*Includes*	*n*
Nursing & midwifery	Nursing unit manager, Registered nurse, Enrolled nurse, Clinical nurse educator, Clinical nurse specialist, Clinical nurse consultant, Midwife (maternity)	26
Medical	Director, Specialist, Registrar, Intern	7
General services	Coordinator, Supervisor, Cleaner, Wards person	5
Administrative	Ward clerk, Clinical support officer	3
Change management	Employees of the hospital change management team	3
Other	e.g., Allied Health Professionals (dieticians, physiotherapists, etc.)	2

**Fig. 1:**
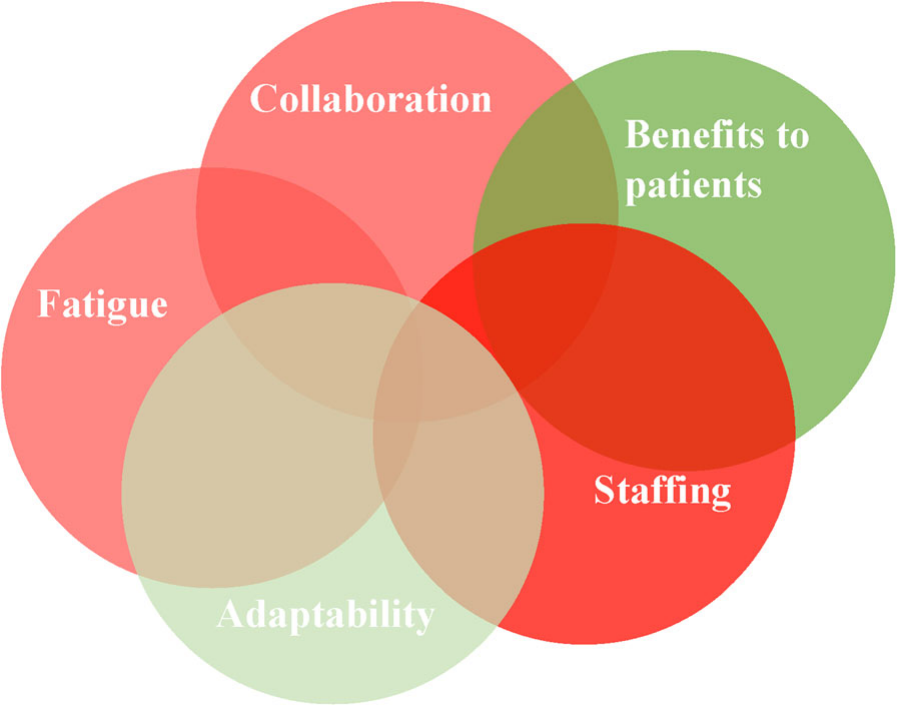
Thematic visualisation of staff understanding and expectations of the change

Five themes were identified related to hospital staff’s understanding and experiences (i.e., expectations and concerns) of the change: staffing; benefits to patients; collaboration; fatigue; and adaptability. These expectations and concerns are schematically presented in Fig. 1, with shades of red indicating negative expectations and concerns associated with the theme, and green representing positive expectations. Intensity of the colour demonstrated the frequency of positivity or negativity associated with that theme (i.e., deeper shades of red indicate frequency of negative discussion of this theme by different hospital staff). This figure also highlights the complexity and interrelatedness of these themes (e.g., the concern of inadequate staffing for the new building was linked with concerns about patient care, which could possibly impede the way the team work together, leading to staff feeling overworked and worn out; these expectations were all mitigated by the staff member’s understanding and awareness of the change). Explanations and examples are presented below.

### Staffing

Hospital staff consistently held staffing to be a major concern in this redevelopment. To them, the opening of the new building, and with it the increase in physical size and addition of new services, meant that an increase in staff was crucial to successfully implement the change: “*My biggest uncertainty at the moment is the fact that I’m really concerned about whether I’m actually going to get enough staff*” (GS1). Many participants suggested that this issue would determine the success of the new hospital building. This was particularly important for staff moving into the new building with a bigger work space: “*We just need more staff. Yeah I think that’s the main issue - if we fix that then I believe everything should be smooth*” (ICUN4). For the most part, staff were unaware about how many new staff they would have in the new building. This uncertainty involved two related issues: (1) will we get the budget for new staff that we need? And if so, (2) where will we find all these new staff to employ?

On the first point, staff reported concerns that they would not have enough staff to cover the increased physical space and new ways of working within the new building. This lingering uncertainty was the result of external factors, specifically unresolved budget issues: “*But I suppose some of the issues stem from the fact that you never know how many beds we are able to open based on the funding from the government, and that is what is still up in the air*” (ICUDR1).

Regarding the second point, staff noted that even if budgetary issues were resolved, and there was enough money to hire new staff to fill the new building, a challenge would be finding the staff to recruit: “*I don’t know where these new staff are going to come from”* (GN3). Some participants suggested that they already encountered difficulties with employing enough appropriately qualified staff and reported concerns that this issue would be compounded when they moved into the new building: “*Excitement will be way gone. It’s more to deal with that stress and the workload of other staff*” (ICUN4). Participants working on wards that were not moving into the new building also reported concerns about staffing. They noted that, despite not being directly involved in previous stages of the redevelopment, they had still been affected by these changes, because their colleagues were taken from their ward without consultation and moved into a new area. Hence, even staff not moving in the next stage of the redevelopment had concerns that their staffing levels would be affected: “*We have been told that we are not moving in there. And hopefully they don’t take our staff there*” (SURN5).

### Benefits to patients

Many hospital staff expressed a positive expectation of the move related to benefits for patients. This was consistent across wards, departments and professions. Staff expected patients to experience benefits including reductions in infection rates and improved satisfaction, due to staying in a well-controlled and physically appealing environment with natural light: “*Any new place will give some joy or some happiness to people… The major change will be that because there are individual rooms, the infection rate will be lower and that I’m very pleased with”* (ICUDR1).

Despite these participants reporting the improved physical environment was expected to positively affect patients, they also raised concerns that being in the new building might negatively affect patient safety because the increased physical space could introduce more room for error with the greater workload: “*Brings with it the fear, of how will we treat so many patients with nursing when you have one to one and the rooms are closed. That is a constant worry*” (ICUDR1). Participants indicated that this issue would be compounded if staffing levels were not increased.

### Collaboration

Staff expressed multiple negative expectations or concerns about how their ways of working together would be affected by moving into the new building. Staff understood the change as more than just a physical expansion, but as an organisational change that would affect their ways of working. This understanding led to concern regarding how to work together in the new building. Specifically, staff moving into the new building were worried about the new layout of ICU, where nurses would be working alone in rooms with single patients. This would disrupt their ability to easily ask for support currently done by asking the nurse at an adjacent bed, or signalling to someone visible across the room: “*Single rooms are great for patients and everything but I think it becomes a bit more isolated for staffing*” (ICUOTH1). These concerns were also recognised among staff working in the change management team, who may not be directly affected by the change, but acknowledged that this is a major consequence of the move into the new building: “*All the beds, they were able to see each other all the time whereas now it’s a different work environment. They’re a bit more isolated… So that’s what we find is the challenge”* (CHGTEAM2). Further, staff were concerned about working in open plan spaces that limit opportunity for private discussions, for example with other staff about workplace conflict or personal matters: “*I’m very concerned about insufficient space for private stuff*” (ICUAD1).

Staff reported negative expectations of collaboration breakdown not only within wards, but across the hospital. The organisational change will include far-flung staff and expanded infrastructure, which may decrease opportunity to collaborate directly. For several participants, the growing size of the hospital was seen as a fracturing of the positive, cohesive culture of what was once a smaller hospital—“*It used to be that the general manager would walk through and know everybody by name, the cleaner, maintenance crew, everybody knew everybody’s name*” (GN1)—into more disconnected, subunits: “*Now we’re very separate*” (ICUOTH1).

### Fatigue

During interviews, many participants reported feeling over-worked and under-resourced. While some described being fatigued and unhappy at work, the redevelopment was, nevertheless, clearly a positive: “*We’re not happy because we’re under so much pressure and stress. But, you know, we are looking forward to the new build, it’ll be a beautiful building”* (GN3). For others, there were concerns that their feelings of being over-worked would not subside with the opening of the new hospital building and that there was a lack of time to even consider the change. This was expressed by staff moving in to the new building, as well as those not moving:

*Who has got the time to go and look at those decorative things*! (SURN5).

*I can’t see how it will make a big difference to me… I don’t pay a lot of attention to the looks* (MATDR1).

*It doesn’t really matter… I could be providing it [patient care] in a tent or a building*. (MATMW2).

Further, hospital staff expressed frustration in having to endure poor resourcing, which tempered their excitement for the new building: *“We’ve all put up with whatever since whenever and I’m done, I’m so done”* (ICUAD1). Some participants reported negative expectations related to the increase in physical space in the new building, as adding to the work load of clinical staff and requiring they travel further to get supplies and attend to patients: *“They are worried about, hang on I’m going to have to do so many more laps”* (ICUAD1). Similarly, an issue expressed on behalf of staff in the General Services Department was whether they will be able to adequately clean and cater for physically larger areas: “*I’m sitting here and looking at* [a previous building that was opened] *and seeing how filthy it is*” (CHGTEAM3). Concerns about being over-worked in the face of the redevelopment were further emphasised by some interviewees who discussed a problem with turnover: “*We’ve actually had a few people, I have had three people, which is unusual for us, who have looked for other jobs and are probably resigning. You know which is sort of the opposite of what we’d expect at this time, we’d expect they’d be excited for the new building*” (ICUN5). However, most staff in more junior positions had not seen the new building and thus were unaware of the layout and the degree to which it may impact their work: “*Because I have not seen the actual structure of the area, and I don’t know what they based it on and how they figured out a way to be friendly for both staff and patients at the same time*” (ICUN3). The unawareness and lack of understanding accentuated concerns and negative expectations among staff as they expected the worst.

Also contributing to reports of experiencing fatigue, staff described numerous other large changes taking place at the hospital over the years, in addition to the redevelopment: “*Basically for seven years we’ve been undergoing changes since I’ve been here. It is utterly exhausting having this many changes all the time*” (GS1). This highlights that while this study captures prospective insights to the change, change is constant in health care. While the move into the new building has not yet occurred, the move is part of a broader organisational change grander than the physical expansion of infrastructure. While this was a major concern for many staff, some of the senior medical staff dismissed this as being an issue, suggesting constant change is part of health care and should not lead to staff feeling worn out: “*I think once you get to my level you get good at kind of jumping through hoops… As you get more experienced, you just go with the flow a bit more”* (SURDR2).

### Adaptability

An additional theme involved staff’s positive expectation that they would be able to adapt to the changes brought about by the move into the new building. Reflecting on past experiences of organisational and infrastructure changes at the hospital, staff expressed that it could take time to adapt and see the benefits of the change: “*At the beginning, of course, everybody was scared of the changes and stuff like that, but eventually we got used to it.*” (SURN3). However, some staff reported that they saw adapting to the new building as a concern, potentially because of a lack of knowledge pertaining to what the new building entails: “*I just don’t know. I’m worried because I don’t know what we’re walking in to*” (ICUN2). In general, staff expressed an understanding of the change as one of physical growth (hospital redevelopment) and changes in ways of working (organisational change): *“Getting bigger. So, basically taking all of our acute services and putting it in a brand spanking new building where they’re significantly expanding”* (GN1); “*The biggest change is changing the way they work. Changing the way they deliver care*.” (CHGTEAM2). When asked why the change was happening, hospital staff were consistent in attributing the need for redevelopment to population growth: “*To develop more resources to accommodate for the growing number of patients*” (SURDR3).

Feeling uninformed and uncertain about the change was expressed by staff of different professions and different levels throughout the hospital. In fact, even wards that were not moving to the new building were unsure if this was the case: “*There’s been no communication from anyone really. I hear from different people yes we are moving and then somebody says no we’re not. We’re staying here in the old building. So, I’m not sure exactly who’s going”* (SURN1).

## Discussion

Our findings suggest that in the early stages of hospital redevelopment, staff experience both positive and negative expectations that are dependent upon the level of personal understanding, awareness of the change to come, and how well-resourced they already feel. Interviews with hospital staff highlighted a general understanding of the change as involving physical expansion of the hospital. However, participants also reported feeling inadequately informed about what is to come and described a range of sometimes differing expectations about the organisational effects of this change (e.g., on collaboration, for patients). This supports the conceptualisation of hospital redevelopment as not only a physical change, but an organisational one too.

The present study is the first to empirically explore the experiences and understanding of staff in the early stages of a hospital redevelopment, and conceptualised this as an organisational change. This conceptualisation is an important contribution to the organisational change literature because we show that change, even when based on the best evidence-based design, can be disappointing and bring about negative experiences for staff. The concerns and negative expectations of the change expressed by staff in the present study echo past research that retrospectively explored the experiences of staff during a hospital change, in Australia [13], and elsewhere [e.g., 14]. In the present study, staffing was a major concern reported by hospital staff. This is consistent with other reports of hospital redevelopment in the Australian context. For example, in a report into the opening of a new children’s hospital, staff were frustrated about the progression of the change and that a lack of staffing impacted on service planning. Staffing was also emphasised as an issue in another Australian hospital redevelopment project, where the building opened with insufficient staffing and resources [27]. Additionally, hospital staff in the present study indicated that they felt fatigued, so much so that excitement for the opening of the new building was diminishing. Reports of low staff morale in hospital redevelopment projects has also been documented in other Australian and international studies [13, 14]. Further, participants in this study reported a lack of awareness of the redevelopment, something that appears to be common with a report of hospital revitalisation in the United States reporting a similar finding [28].

One source of many of the issues expressed by staff was uncertainty, a common and often inevitable experience in health care [29], for example, systems uncertainty about staffing levels and uncertainty about whether collaboration and support would break down as the hospital expands. While some types of uncertainty cannot be eradicated, it is important to manage uncertainty in times where information is available. One way to do this is to make sure front-line actors have a platform to seek information and ask questions during organisational change; having access to information is a predictor of success for organisational change in healthcare [30]. This may help alleviate stress associated with change and make the transition period less uncertain for staff, particularly in early stages where uncertainty may be greater. While it is not always possible for all the concerns and expectations of staff to be individually acknowledged and addressed by those coordinating the change (e.g., change management team or hospital executives), an alternative is through the use of ‘champions’ or ‘opinion leaders’. Opinion leaders are actors with a brokerage role; they carry information across social boundaries, such as between groups of professionals or different hospital wards [31]. Otherwise referred to as a ‘champion’, by virtue of their trustworthiness and connectedness, these actors are able to lead the opinions of others and are integral in the adoption and diffusion of new phenomena. Successful champions are enthusiastic and motivated about the change they are promoting [32]. In this case, a successful champion in a hospital undergoing organisational change is a staff member who can inform others and influence acceptance, and provide a positive frame for the change.

### Implications

While findings may be localised to the hospital we researched, it is important to note that the hospital redevelopment under investigation is similar to other hospital redevelopments in metropolitan cities in Australia [7] and worldwide [5]. Specifically, the redevelopment is an expansion of infrastructure to meet the growing needs of the community which the hospital serves. The perceptions and experiences maintained by hospital staff will differ dependent on the state of the new facilities; these findings broadly generalise to any hospital redevelopment where a newer, larger building is opened. The implications of this study provide broad suggestions for other hospitals undergoing this type of hospital redevelopment.

Firstly, hospital redevelopment should be considered as more than physical change, but as an organisational change, in order to recognise the ripple effects of changing the infrastructure and how this may influence social and behavioural processes. From this study’s findings of the expectations and present experiences of organisational change, we recommend four strategies to aid in the early stages of hospital redevelopment: engage actors; plan and train; learn from the past; and increase managerial engagement (see Table 2). These recommendations correspond with suggestions from a past review examining transforming systems in health care [33]. Effort must be taken to ensure staff are informed of the change and rectify any confusions about who, what, when, and how the change is taking place. This is consistent with organisational change theory that maintains that large scale change requires significant effort and planning to ensure its success [19]. Therefore, an implication of this study lies in the importance of exploring the understanding and expectations of staff preceding a large organisational change in order to aid in the acceptance of, rather than resistance to, the change [21]. Further, this study also highlights the importance of studying the experiences of actors not directly involved in the organisational change but who are a part of the broader system (i.e., wards not moving implied they will be affected).

**Table 2:** Recommendations for organisational change in hospitals

		Explanation	Exemplar quote
1.	Engage actors	Staff, patients, families, community members should be engaged in the move. For example, certain staff can be used as ‘champions’ to address concerns and inform co-workers about the new build.	*“I was involved in this part of the build, so it gives a bit of ownership to the new unit”*
		This may foster commitment, a sense of ownership, renewed motivation, engagement in work and excitement that may aid in the smooth transition and adoption of the new build.	*“We need people who are keen, committed and positive about the move and able to support staff in that transition.”*
2.	Plan and train	Hospital staff suggested that having a concrete plan and training staff for the new facilities is essential. Planning and training will give staff the opportunity to acclimatize to the new environment and reflect on how it will change their ways of working	“*I think it’s really important that we be organised so that staff are well oriented and ready for the move rather than just moving and staff are not ready for it”*
3.	Learn from the past	This hospital redevelopment is part of a multi-stage project similar to other hospitals. Hospital staff highlighted that individuals running this project must learn from the issues that occurred in the first stage of the redevelopment, as well as the experiences of other hospitals.	“*I’ve been across to […] and spoken to them because they’ve come from a very similar thing… Just looking at what challenges they had… learn from their experiences*.”
4.	Increase managerial engagement	Hospital staff in more junior positions expressed a lack of collaboration with executives and a lack of communication regarding elements of the redevelopment process. There was a general sense of disconnect between hospital management and staff, expressed by front-line clinicians.	*“I think there probably needs to be more collaboration from above, as in more information because it’s like this is what’s happening. And you’ve had no involvement in the process. I think that’s wrong.”*

### Strengths and limitations

A strength of this study lies in the number of participants and variability in the professions that contribute to the transferability of the study findings. Further, checking and clarifying themes with other researchers throughout the coding process increases the trustworthiness of the findings [26]. As to limitations, interviews were on average 17 min long, with the shortest interview lasting seven minutes. While this may be perceived as a short duration for collecting interview data it was appropriate for participants who were incredibly time poor (e.g., nurses on shift who could only get a 10 min break to talk to the researcher). It is important that the opinions of these busy staff are captured to reflect the true nature of a sample of varied hospital staff. Further, the findings may not be generalisable to other instances of organisational change and may be specific to the four wards and hospital examined in this study. Wards were purposively chosen rather than randomised. While findings may be specific to the hospital under investigation, the research has been designed to optimise research credibility in this qualitative analysis. Further, considerable context was provided to help readers infer relevance to different settings. This in-depth analysis of how staff understand and interpret organisational change in hospitals provides the opportunity to uncover theoretical insights into the processes of change in the health care system and the perspectives of staff during times of organisational change.

## Conclusions

This study explored the prospective understanding and experiences of staff in organisational change in hospitals, using an Australian hospital redevelopment as a case exemplar. Findings indicated that staff were concerned about staffing levels, fatigue, and the potential for a breakdown of current collaborative working. These concerns are similar to past reports of redevelopment in hospitals. This paper presents recommendations for the early stages of organisational change in hospitals. For present and future hospital organisational change projects, it is important that staff concerns are addressed and that staff are informed adequately about the ongoing changes in order to improve their engagement and ownership of the change.

## Supplementary information

**Additional file 1.** Consolidated criteria for reporting qualitative studies (COREQ): 32-item checklist.

**Additional file 2.** Semi-structured interview guide.

## Data Availability

The datasets analysed during the current study are not publicly available due to individual privacy, but are available from the corresponding author on reasonable request.

## Abbreviations

AD: Administrative staff
CHGTEAM: Change management team staff
DR: Medical staff
G: General – works across several wards
GS: General services staff
ICU: Intensive care unit
MAT: Maternity ward
MW: Midwifery staff
N: Nursing staff
OTH: Other profession
RES: Respiratory ward
SUR: Surgical ward
