# Supplementary material for: Organisational change in hospitals: a qualitative case-study of staff perspectives
Source: BMC Health Serv Res. 2019 Nov 14;19:840. doi: 10.1186/s12913-019-4704-y (PMC6857127; doi:10.1186/s12913-019-4704-y)
Supplement: Supplementary file 1 — Additional file 1. Consolidated criteria for reporting qualitative studies (COREQ): 32-item checklist. [file 12913_2019_4704_MOESM1_ESM.pdf]

Supplementary File A – Consolidated criteria for reporting qualitative studies (COREQ): 32-item checklist

Tong A, Sainsbury P, Craig J. Consolidated criteria for reporting qualitative research (COREQ): a 32-item checklist for interviews and focus groups. *International Journal for Quality in Health Care*. 2007. Volume 19, Number 6: pp. 349 – 357

No. Item	Guide questions/description	Reported on Page #
Domain 1: Research team and reflexivity		
Personal Characteristics		
**1. Inter viewer/facilitator**	Which author/s conducted the interview or focus group?	Page 8
**2. Credentials**	What were the researcher's credentials? E.g. PhD, MD	Page 1 and 8
**3. Occupation**	What was their occupation at the time of the study?	Page 1 and 8
**4. Gender**	Was the researcher male or female?	Page 1
**5. Experience and training**	What experience or training did the researcher have?	Page 1 and 8
Relationship with participants		
**6. Relationship established**	Was a relationship established prior to study commencement?	Page 8
**7. Participant knowledge of the interviewer**	What did the participants know about the researcher? e.g. personal goals, reasons for doing the research	Page 8
**8. Interviewer characteristics**	What characteristics were reported about the interviewer/facilitator? e.g. Bias, assumptions, reasons and interests in the research topic	Page 8
Domain 2: study design		
Theoretical framework		
**9. Methodological orientation and Theory**	What methodological orientation was stated to underpin the study? e.g. grounded theory, discourse analysis, ethnography, phenomenology, content analysis	Page 8
Participant selection		
**10. Sampling**	How were participants selected? e.g. purposive, convenience, consecutive, snowball	Page 7
**11. Method of approach**	How were participants approached? e.g. face-to-face, telephone, mail, email	Page 7 and 8
**12. Sample size**	How many participants were in the study?	Page 9
**13. Non-participation**	How many people refused to participate or dropped out? Reasons?	Page 7
Setting		

**14. Setting of data collection**	Where was the data collected? e.g. home, clinic, workplace	Page 7
**15. Presence of non-participants**	Was anyone else present besides the participants and researchers?	Page 7-8
**16. Description of sample**	What are the important characteristics of the sample? e.g. demographic data, date	Page 6 and 7
Data collection		
**17. Interview guide**	Were questions, prompts, guides provided by the authors? Was it pilot tested?	Yes. Page 8 and Supplementary File B
**18. Repeat interviews**	Were repeat interviews carried out? If yes, how many?	No.
**19. Audio/visual recording**	Did the research use audio or visual recording to collect the data?	Yes. Page 8
**20. Field notes**	Were field notes made during and/or after the interview or focus group?	No. Page 8
**21. Duration**	What was the duration of the interviews or focus group?	Page 9
**22. Data saturation**	Was data saturation discussed?	Yes. Page 7
**23. Transcripts returned**	Were transcripts returned to participants for comment and/or correction?	No. Page 8
Domain 3: analysis and findings		
Data analysis		
**24. Number of data coders**	How many data coders coded the data?	Page 8
**25. Description of the coding tree**	Did authors provide a description of the coding tree?	Page 9 and Figure 1
**26. Derivation of themes**	Were themes identified in advance or derived from the data?	Page 8
**27. Software**	What software, if applicable, was used to manage the data?	Page 8
**28. Participant checking**	Did participants provide feedback on the findings?	No. Page 8.
Reporting		
**29. Quotations presented**	Were participant quotations presented to illustrate the themes/findings? Was each quotation identified? e.g. participant number	Yes. Page 10 - 15
**30. Data and findings consistent**	Was there consistency between the data presented and the findings?	Yes. Page 10-15
**31. Clarity of major themes**	Were major themes clearly presented in the findings?	Yes. Page 10-15
**32. Clarity of minor themes**	Is there a description of diverse cases or discussion of minor themes?	Yes. Page 10-15
