# Supplementary material for: Organisational change in hospitals: a qualitative case-study of staff perspectives
Source: BMC Health Serv Res. 2019 Nov 14;19:840. doi: 10.1186/s12913-019-4704-y (PMC6857127; doi:10.1186/s12913-019-4704-y)
Supplement: Supplementary file 2 — Additional file 2. Semi-structured interview guide. [file 12913_2019_4704_MOESM2_ESM.pdf]

Supplementary File B – Semi-Structured Interview guide

Introductory Questions:

- Can you please tell me about your role at XXX Hospital?
- How long have you worked at XXX Hospital?

Enquiry into Hospital Expansion:

- In your own words, what are the changes going on at XXX Hospital?
- Why do you think this change is happening?
- What do you think will come as a result of this large hospital change?
- Are you uncertain about any of these changes?

Personal Impact:

- Do you think these changes will impact your role? (positive, negative, or unexpected)
    a) Do you have any concerns about how the hospital redevelopment project will affect you?
    b) What are you most looking forward to in regards to the changes?

Culture & Interprofessional Collaboration:

- With the transition into the new building, do you think there will be a change in the way staff work together?
    a) Hospital level
    b) On the ward you work on
- Do you think there will be a change in culture?

Concluding Remarks:

- Is there anything else you would like to add?

That is the end of the interview. Thank you for your time.
